# An Aloe-Based Composition Constituting Polysaccharides and Polyphenols Protected Mice against D-Galactose-Induced Immunosenescence

**DOI:** 10.1155/2024/9307906

**Published:** 2024-03-14

**Authors:** Mesfin Yimam, Teresa Horm, Alexandria O'Neal, Ping Jiao, Mei Hong, Qi Jia

**Affiliations:** Unigen Inc., 2121 South State Street, Suite #400, Tacoma, WA 98405, USA

## Abstract

A decline in immune response, exhibited in the form of immunosenescence and inflammaging, is an age-associated disturbance of the immune system known to predispose the elderly to a greater susceptibility to infection and poor vaccine response. Polysaccharides and polyphenols from botanicals are known for their immune modulation effects. Here we evaluated a standardized mushroom-based composition, UP360, from *Aloe barbadensis*, *Poria co*cos, and *Rosmarinus officinalis*, as a natural nutritional supplement for a balanced immune response in an accelerated aging mouse model. Immunosenescence was induced by continual subcutaneous injection of D-galactose (D-gal) at a dose of 500 mg/kg/day to CD-1 mice. UP360 was administered at oral doses of 200 and 400 mg/kg to the mice starting on the 5^th^ week of D-gal injection. The study lasted for a total of 9 weeks. All mice were given a quadrivalent influenza vaccine at 3 *µ*g/animal via intramuscular injection 14 days before the end of the study. A group of D-gal-treated mice treated at 400 mg/kg/day UP360 was kept without vaccination. Whole blood, serum, spleen homogenate, and thymus tissues were used for analysis. UP360 was found to improve the immune response as evidenced by stimulation of innate and adaptive immune responses, increase antioxidant capacity as reflected by augmented SOD and Nrf2, and preserve vital immune organs, such as the thymus, from aging-associated damage. The findings depicted in this report show the effect of the composition in activating and maintaining homeostasis of the immune system both during active infections and as a preventive measure to help prime the immune system. These data warrant further clinical study to explore the potential application of the mushroom-based composition as an adjunct nutritional supplement for a balanced immune response.

## 1. Introduction

Immunosenescence and inflammaging are age-associated changes of the immune system that predispose the host to an increased susceptibility to infection and poor vaccine response [1]. Advanced age impacts both innate and adaptive immunity, such as through alterations in immune cell count and function. Despite functional downregulation of the immune response at the cellular and humoral levels, there is a progressive increase in the levels of proinflammatory cytokines and chemokines in the systemic circulation, resulting in low-grade chronic inflammation [2]. This lack of immune regulation warrants the need for restoration of a balanced immune response in the elderly. However, consideration has to be taken to avoid shifting the balance to over or under stimulating the immune system, as over stimulation of the immune system, as in the case of cytokine storm, could potentially cause serious harm to the host [3, 4]. Unlike over stimulation, in the case of a compromised immune response, subjects could easily be susceptible to infection from invading pathogens and experience lower vaccine response rates due to incompetent immune responses [5]. There is a need for a balanced immune response to create immune homeostasis to equilibrate the immune system similar to the normal physiological conditions. Botanicals that possess the ability to activate the innate and adaptive immune responses while mitigating systemic inflammatory response and oxidative stress may have potential use in restoring a balanced immune response. Here, we evaluated an Aloe-based composition, UP360, comprised of polysaccharides and polyphenols from the traditionally well-known medicinal plants *Aloe barbadensis*, *Poria cocos*, and *Rosmarinus officinalis*, for its innate and adaptive immune stimulation, anti-inflammatory, and antioxidation activities in the D-galactose-induced accelerated aging mouse model.

Studies have shown that prolonged subcutaneous injection of D-galactose (D-gal) to mice increased production of free radicals, decreased antioxidant enzyme activity, increased advanced glycation end products, and induced poor immune responses. These changes are similar to observations in the normal aging process, which are linked to the pathogenesis of many age-associated pathologies [6, 7]. This model has been significantly utilized in the field of herbal medicine for benefit assessment. For example, using the D-galactose-induced model: (1) resveratrol prevented thymus involution and recovered immune function [8], (2) ginseng stem-leaf saponins showed mitigated oxidative stress and improved inflammatory responses [9], (3) *Ganoderma lucidum* Rhodiola prevented immune impairment and oxidative stress [10], (4) *Lycium barbarum* polysaccharides alleviated lipid peroxide damage [11], and (5) Yulangsan polysaccharide improved redox homeostasis and immune impairment [12]. The D-gal model is ideal for testing botanicals for improvement in immune function.

Polysaccharides from *A. barbadensis (Aloe vera*) and *P*. *cocos*, and polyphenols from Rosemary (*R. officinalis*) have been identified as lead bioactives in these medicinal herbs and have been used to promote human health for centuries. For example, *A. barbadensis* M. (*A. vera*), has been indicated for wound healing, antimicrobial and anti-inflammatory activities, immune stimulation, skin protection, and hair growth [13, 14]. Acetylated mannan (or Acemannan) is the predominant active polysaccharide component of *A. vera* gel isolated with molecular weights in the range of 3,000–2,000,000 Da [15]. *P. cocos* Wolf is a medicinal mushroom with the major active component of polysaccharides (PCP), in the form of *β*-glucan, with a *β*-(1→3)-linked glucose backbone and *β*-(1→6)-linked glucose side chains. Variable biological functions have been reported for *P. cocos* polysaccharides including antioxidation, anti-inflammation, anticancer, and immunological modulation [16, 17]. Rosmarinic acid (RA), a water-soluble caffeoyl phenolic acid compound, has been reported as one of the principal components in *R. officinalis*. A wide range of biological activities, including antioxidant, antimicrobial, antiviral, anticancer, antiapoptotic, and anti-inflammatory effects, has been reported [18–20]. In the current study, D-galactose-induced accelerated aging model has been utilized to evaluate the immune stimulatory effect of UP360.

## 2. Materials and Methods

### 2.1. Composition

The fresh leaves of *A. vera* plant were washed, and the outer rinds were removed. The whole leaf gel was treated with cellulase enzyme and filtered through activated charcoal. The filtrates were concentrated by low pressure evaporation and dehydrated to dry power by Qmatrix® processing. *A. vera* leaf gel powder was produced in the form of the lyophilizate at an extraction ratio of 200 : 1 with no less than 10% polysaccharides. The molecular weight distribution of polysaccharides was between 50–200 kDa with an average molecular weight of 80 kDa. Ground *P. cocos* powder was extracted with ethanol and water to give an ethanol precipitate with no less than 20% polysaccharides at an extraction ratio of 15–18 : 1. Rosemary leaf extract was manufactured by ethanol/water extraction to give no less than 30% RA at an extraction ratio of 100 : 1.

*A. vera* leaf gel powder, *P. cocos* extract, and Rosemary leaf extract were blended at a ratio of 3 : 6 : 1 by weight to give the final composition of UP360. This ratio was determined following a series of combination studies. Standardized UP360 contains no less than 10% total polysaccharides from both Aloe gel powder and *P. cocos* extract and not less than 2% RA from Rosemary leaf extract.

### 2.2. Increased PBMC Cytotoxicity by UP360 and Its Constituents

K562 lymphoblasts (ATCC, CCL-243) were prestained with Calcein-AM (Millipore Sigma, 206700) for 30 min at 37°C, then were centrifuged and resuspended three times in fresh media to wash out unbound Calcein-AM. Stained K562 cells were plated at 10,000 cells per well in black 96-well cell culture dishes with optical flat bottoms (Fisher Scientific, 50-308-76). PBMCs (Millipore Sigma, 0002145) were added at a 25 : 1 ratio of PBMCs to K562s. Treatments were dissolved in water and administered to PBMCs overnight before incubation with K562 cells. Treatments were added at the following concentrations: UP360 at 25 *µ*g/mL, Aloe extract at 7.5 *µ*g/mL, Poria extract at 15 *µ*g/mL, and Rosemary extract at 2.5 *µ*g/mL. The plate was centrifuged at 500 rpm for 1 min (42 RCF) to settle suspended cells to the bottom of the wells. The plates were scanned using an ImageXpress Pico (Molecular Devices, product: IMAGEXPRESS PICO EC) automated cell imaging system at baseline, and every hour for 4 hr. The number of live cells was analyzed for each well by adjusting the detection parameters for fluorescence intensity and cell diameter. The percent cytotoxicity for each treatment was calculated as the difference in live cells at each time point subtracted from the baseline count, divided by the baseline count. The percent cytotoxicity beyond the normal control was calculated as the difference in percent cytotoxicity between the treatment and the normal control.

### 2.3. D-Galactose Induced Accelerated Aging Model

Purpose-bred CD-1 mice (12 weeks old) were purchased (Charles River Laboratories, Inc., Wilmington, MA) and used for the study after 2 weeks of acclimation. Mice were housed in a temperature-controlled room (71–72°F) on a 12-hr light–dark cycle and provided with feed and water *ad libitum*. Mice were randomly assigned to four immunized groups and three nonimmunized groups. The immunized groups included G1 = normal control + vehicle (0.5% CMC), G2 = D-gal + vehicle, G3 = D-gal + UP360 400 mg/kg, and G4 = D-gal + UP360 200 mg/kg. The nonimmunized treatment groups included G1 = normal control + vehicle (0.5% CMC), G2 = D-gal + vehicle, and G3 = D-gal + UP360 400 mg/kg. While 10 animals were allocated in each treatment group for the immunized set, eight animals were included in each group for the nonimmunized set.

Mice were injected with D-gal at 500 mg/kg subcutaneously daily for 9 weeks to induce accelerated aging. At the end of the 4^th^ week of induction, treatment with two doses of UP360 (200 mg/kg-low dose and 400 mg/kg-high dose) suspended in 0.5% CMC orally commenced. One additional group of UP360 at 400 mg/kg was included to be used as a control for nonimmunized mice. In the 7^th^ week, each mouse except those mice in nonimmunized groups was injected with 3 *μ*g of Fluarix quadrivalent IM (Lot # UJ477AA, 2020–2021) influenza seasonal vaccine from SANOFI. It contained 60 *μ*g hemagglutinin—HA per 0.5 mL single human dose. The vaccine was formulated to contain 15 *μ*g of each of four influenza strains such as H1N1, H3N2, B-Victoria lineage, and B-Yamagata lineage for immunization at a single dose.

Daily oral gavaging of UP360 consisting of polysaccharides and polyphenols for the duration from the 4^th^ week to the 9^th^ week was carried out. At the time of necropsy, (i.e., 14 days after immunization), blood (1 mL) was collected and aliquoted—110 *μ*L for a flow cytometry immunity panel in BD Microtainer® Tubes with Dipotassium EDTA (Lavender), which were delivered on ice to Flow Contract Site Laboratory (Bothell, WA), serum was isolated from the remaining blood (SST, centrifuged at 1,500 rpm for 10 min at 4°C, about 400 *μ*L serum yield) for antibody ELISAs and enzymatic assays. Weights of the thymus for each animal were taken to determine thymus indices. The spleens were kept on dry ice at the time of necropsy and transferred to −80°C for future use.

### 2.4. Senescence-Associated *β*-Galactosidase Staining

At necropsy, the thymus was dissected from each mouse and fixed in prechilled 4% paraformaldehyde for 24 hr before it was transferred to a 30% sucrose solution for an additional 24 hr. Fixed tissues were then snap frozen in liquid nitrogen and shipped to Nationwide Histology (Missoula, MT), packed in dry ice, for analysis. Tissues were flash frozen in cryoprotectant and sectioned at 10-micron thickness onto Superfrost Plus slides. Tissues were then rinsed in phosphate buffered saline (PBS; 0.05 M PO_4_ and 0.9% NaCl, pH 7.4) and the protocol for the *β*-galactosidase staining kit (product # 9860) from Cell Signaling Technologies was followed. A light eosin counterstain was added for contrast and slides were mounted with nonaqueous mounting medium (Sigma, DPX 1.00579). The senescent cells were then counted in quadrants to determine the overall percentage of positive cells. An Olympus BH2, Nikon Eclipse 800 microscope with an Olympus DP26 camera operating with cellSens Standard 1.9 software was used for cell counting and imaging.

### 2.5. Serum SOD Measurement Method

Mouse serum samples were tested for superoxide dismutase (SOD) levels using a superoxide dismutase assay kit (Cayman Chemical, 706002) as follows: serum was diluted 1 : 5 in assay buffer before assay. 10 *μ*L of sample was added to each well along with Radical Detector (tetrazolium salt used as a colorimetric indicator of free radical content). 20 *μ*L xanthine oxidase was added to generate free radicals from hypoxanthine and oxygen. The plate was incubated on a plate shaker for 30 min at room temperature before the absorbance was read at 450 nm. Each sample was assayed in duplicate and the absorbances of each sample were compared to a superoxide dismutase standard curve to deduce the SOD activity (U/mL) and multiplied by the dilution factor.

### 2.6. Spleen Homogenate Nrf2 Measurement Method

Mouse spleen tissues were homogenized in RIPA lysis buffer (50 mM Tris-HCl, pH 7.6, 150 mM NaCl, 1% NP-40, 0.5% sodium deoxycholate, 0.1% SDS) at a concentration of 100 mg tissue/mL buffer for 3 × 10 s on ice. The homogenates were then incubated on ice for 30 min to enhance tissue lysis and centrifuged at 16,000 RCF for 10 min at 4°C. The supernatants were transferred to new microcentrifuge tubes and frozen at −80°C. Spleen homogenates were run on SDS-PAGE and blotted using a rabbit anti-Nrf2 (Life Technologies, 16396-1-AP) antibody with a Goat anti-Rabbit IgG Alexa Fluor Plus 488 secondary antibody, and anti-*β*-actin (Life Technologies, MA5-15739-D680).

### 2.7. Flow Cytometry Method

**Figure 1:**
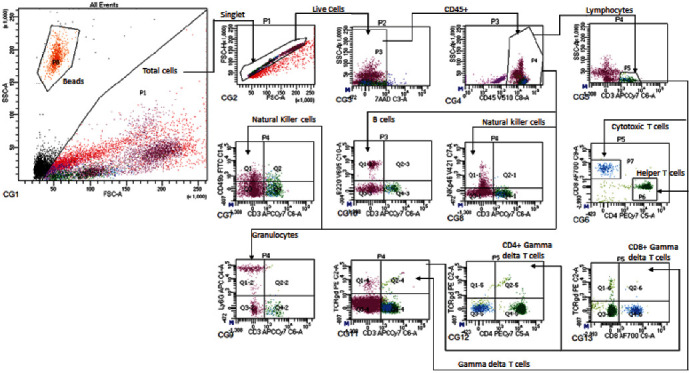
Flow cytometry forward and side scatter gating strategy for immune cells. Whole blood samples were assessed for 10-marker flow cytometry analysis. The total cell population was identified using forward and scattered gating after the exclusion of doublets and debris. The following markers were assessed to determine cell populations, according to marker expression listed in Table 1: CD45, CD3, CD4, CD8, CD49b, B220, NKp45, Ly6G, TCR*γδ*, and 7-AAD.

Whole blood was collected by cardiac puncture, transferred to EDTA blood collection tubes and refrigerated overnight (nonimmunized samples) or nutated at room temperature for up to 6 hr (immunized samples). The whole blood samples were delivered on ice to the Flow Contract Site Lab (Bothell, WA) for 10-marker flow cytometry analysis. Doublets and debris were excluded, and the total cell population was identified using forward and scattered gating. Fluorescent antibodies were then used to identify cell populations (Figure 1 and Table 1). Whole blood was incubated with the following antibodies for 15–20 min: mouse (m)CD45 V510 clone 30-F11 (BioLegend, #103137), mCD3 APCCy7 clone 17A2 (BioLegend, #100221), mCD4 PECy7 clone GK1.5 (BioLegend, #100421), mCD8 AF700 clone 53–6.7 (BioLegend, 100729), mCD49b FITC clone DX5 (BioLegend, #108905), mLy6G APC clone 1A8 (Biolegend, #127613), mCD45R/B220 V605 clone RA3-6B2 (BioLegend #103243), mNKp46 V421 clone 29A1.4 (BioLegend, #137611), mTCR*γδ* PE clone GL3 (BioLegend, #118107), 7-AAD (BD or BioLegend, #420403). Red blood cells were lysed with PharmLyse (BD, #555899) and the samples were centrifuged and resuspended in calcium and magnesium-free DPBS (ThermoFisher Scientific, #J67802K2) before being mixed with CountBright beads (ThermoFisher Scientific, #C36950). Flow cytometric data acquisition was performed using FACSCantoA, FACSCanto 10 Color flow cytometer and BD FACSDivaTM software. Cell populations were determined by electronic gating on the basis of forward versus side scatter.

### 2.8. Statistical Analysis

Data were analyzed using SigmaPlot 14.5 data analysis software (Palo Alto, CA). The results are represented as mean ± SD. Statistical significance between groups was calculated by means of single factor analysis of variance and a paired *t*-test. *P*-values less than or equal to 0.05 (*P* ≤ 0.05) were considered as statistically significant. Percent changes from vehicle were calculated as % changes = {(mean value of vehicle−mean value of active test article)/(Mean value of Vehicle)} × 100.

## 3. Results

### 3.1. Cytotoxicity Induction in PBMCs by UP360 and Its Constituents

**Figure 2:**
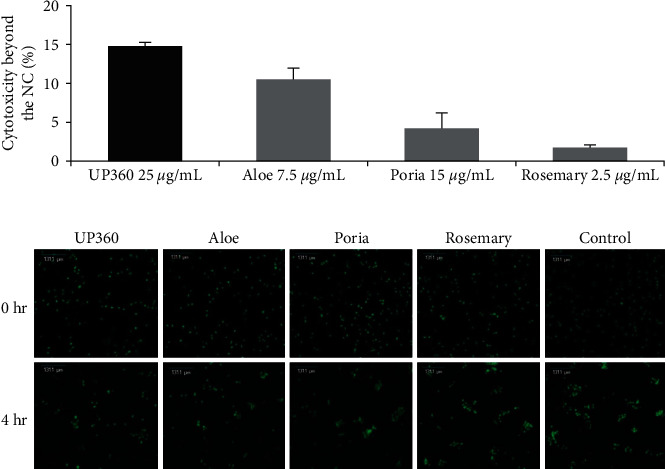
Cytotoxicity effect of UP360 and its constituents. PBMCs were added at a 25 : 1 ratio of PBMCs to K562s, and treated at concentrations of 25, 7.5, 15, and 2.5 *µ*g/mL with UP360, aloe, Poria, and Rosemary, respectively. The plates were scanned using an automated cell imaging system at baseline, and every hour for 4 hr. The number of live cells was analyzed for each well by adjusting the detection parameters for fluorescence intensity and cell diameter. The percent cytotoxicity for each treatment was calculated as the difference in live cells at each time point subtracted from the baseline count, divided by the baseline count. The reported percent cytotoxicity beyond the normal control was calculated as the difference in percent cytotoxicity between the treatment and the normal control: (a) percent cytotoxicity beyond the normal control and (b) representative cell imaging at 0 and 4 hr.

Peripheral blood mononuclear cells (PBMCs) were incubated with K562 tumor cells for up to 4 hr, inducing a cytotoxic effect from the natural killer cells present in the PBMCs that resulted in K562 cell death. Untreated PBMCs exhibit efficient cytotoxicity towards K562 cells, but we tested UP360 and its constituents for enhancement of K562 cell death, or increased cytotoxicity activity of PBMCs UP360 at 25 *µ*g/mL, Aloe extract at 7.5 *µ*g/mL, *P*. *cocos* extract at 15 *µ*g/mL, and Rosemary extract at 2.5 *µ*g/mL (equivalent dose to the UP360 composition), were added to PBMCs and Calcein-AM-labeled K562s for 4 hr. Cytotoxicity of K562 cells was assessed by measuring the number of Calcein-AM-stained cells at T0 compared to T4 hr. The activity of the treatments was represented as additional percent cytotoxicity beyond the negative (vehicle) control. UP360-treated PBMCs exhibited a statistically significant 13.8% increase in cytotoxicity to K562 lymphoblasts beyond the cytotoxicity observed in the negative (vehicle) control. Aloe extract exhibited a statistically significant 9.5% increase in cytotoxicity; Poria extract exhibited a 3.2% increase in cytotoxicity; and Rosemary extract exhibited a 0.77% increase in cytotoxicity (Figure 2). The combined increases of the three extracts totaled to 13.5% increase beyond the cytotoxicity of the normal control, which was comparable to UP360′s 13.8% increase, suggesting the additive effect of these constituents. UP360 cytotoxicity was tested on K562 cells at concentrations up to 1 mg/mL and no cytotoxicity was found from UP360 itself. After a 4-hr incubation with 1 mg/mL UP360, the K562 cells still had 100% viability. UP360 and its constituents effectively increased PBMC cytotoxicity towards K562 tumor cells, demonstrating UP360′s enhancement of the innate immune system.

### 3.2. Effect of UP360 on the Immune Organ: The Thymus

**Figure 3:**
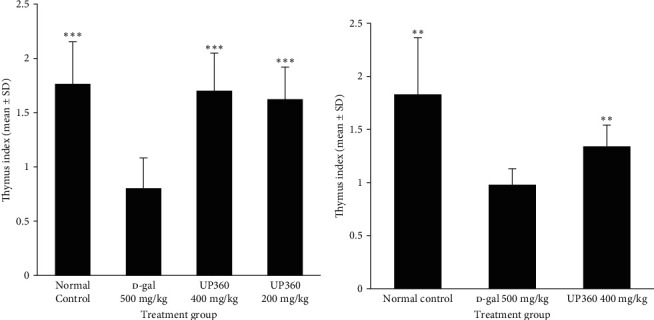
Thymus indices in D-galactose-induced aged mice treated with the Aloe-based composition. Mice were injected with D-galactose at 500 mg/kg subcutaneously daily for 9 weeks to induce accelerated aging: (a) mice (*n* = 10) were treated with two doses of UP360 (200 or 400 mg/kg) orally starting from week 5 and immunized at the start of week 8 with 3 *μ*g of Fluarix quadrivalent vaccine and (b) D-gal mice (*n* = 8) were treated with UP360 at 400 mg/kg orally and compared against the normal control and D-galactose induced mice without vaccination. Necropsy was carried out 14 days after vaccination at the end of week 9.  ^*∗∗*^*P* ≤ 0.001 vs D-gal control;  ^*∗∗∗*^*P* ≤ 0.0001 vs D-gal control.

In the immunized groups, D-gal mice treated with the vehicle showed a significant reduction (54.5%) in the thymus index compared to the normal control mice. This reduction in thymus index was reversed by both dosages of UP360. Mice treated with UP360 orally at 400 and 200 mg/kg showed a 52.9% and 50.6% increase in thymus index, respectively, when compared to the vehicle-treated D-gal group (Figure 3(a)). This reversal was statistically significant compared to vehicle-treated D-gal mice for both doses of UP360. Similarly, the nonimmunized group treated with UP360 at 400 mg/kg also showed a statistically significant increase in the thymus index (Figure 3(b)). This increase was found to be 26.9% when compared to the nonimmunized vehicle-treated D-gal mice.

**Figure 4:**
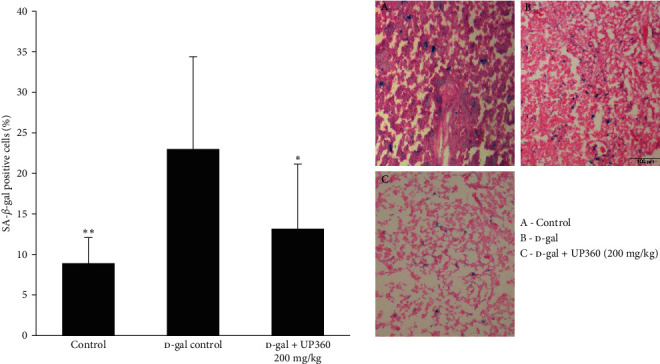
Effect of UP360 on senescence-associated *β*-galactosidase cells. Mice (*n* = 10) were injected with D-galactose at 500 mg/kg subcutaneously daily for 9 weeks to induce accelerated aging. Mice were treated with the Aloe-based composition, UP360, starting week 5 and immunized at the end of week 7 (start of week 8). Necropsy was done at the end of week 9. Thymus tissues collected from mice treated with 200 mg/kg of UP360, normal control, and D-gal control were used for senescence-associated *β*-galactosidase staining.  ^*∗*^*P* ≤ 0.05 vs D-gal control;  ^*∗∗*^*P* ≤ 0.001 vs D-gal control.

Senescence-associated-*β*-galactosidase (SA-*β*-Gal) staining detected senescent cells in each thymus to evaluate the immune organ protective effects of UP360. SA-*β*-Gal positive cells were found stained blue (expressing highly senescence-specific *β*-galactosidase) and randomly scattered throughout the cortex and medulla. The changes observed for the lower dose of UP360 to the thymus histology were in alignment with the thymus index data. Immunized mice treated with UP360 low dose (200 mg/kg) showed a statistically significant reduction in the proportion of senescent cells when compared to the vehicle-treated D-gal mice. Subcutaneous administration of D-gal produced a 157.8% increase in senescent cells when compared to the normal control mice, whereas mice treated with UP360 at 200 mg/kg showed a 42.7% reduction in senescenct cells in comparison to the vehicle-treated D-gal mice (Figure 4).

### 3.3. Effect of UP360 on Circulating Immune Cell Populations in Immunized Mice

**Figure 5:**
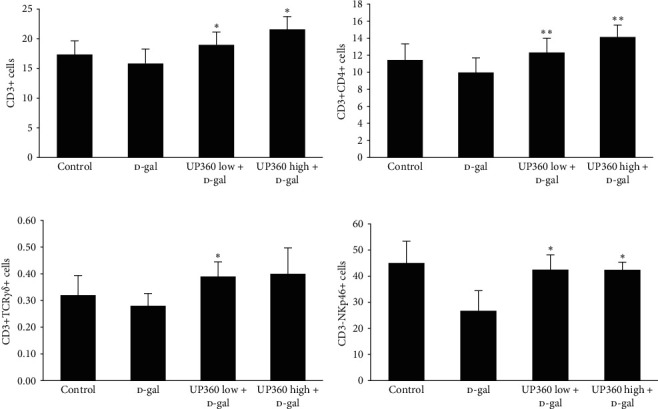
Effect of UP360 on immune cell expansion in immunized mice. Mice (*n* = 10) were injected with D-galactose at 500 mg/kg subcutaneously daily for 9 weeks to induce accelerated aging. Mice were treated with two doses of UP360 (200 or 400 mg/kg) orally, starting at week 5 and immunized at the end of week 7 (start of week 8) with 3 *μ*g of Fluarix quadrivalent vaccine. Necropsy was carried out 14 days after vaccination at the end of week 9. Flow cytometry data are reported as mean ± SD. (a) T cells; (b) helper T cells; (c) Gamma–delta T cells; (d) natural killer cells.  ^*∗*^*P* ≤ 0.05 vs D-gal control;  ^*∗∗*^*P* ≤ 0.001 vs D-gal control.

The levels of circulating immune cells in the immunized mouse groups were assessed at the end of the study. Expressed as a percentage of all white blood cells (CD45+ cells), we found that 2 weeks after influenza vaccination, statistically significant increases in T cells (CD3+CD45+; *P* ≤ 0.05; Figure 5(a)), Helper T cells (CD3+CD4+; *P*=0.001; Figure 5(b)), CD3+TCR*γδ*+ Gamma–delta T (*P* ≤ 0.05; Figure 5(c)) and natural killer cells (CD3−NKp46+; *P*=0.02; Figure 5(d)), were observed for the mice treated with the lower dosage of UP360 when compared with the vehicle treated D-gal group (Table 2). The immunized animals treated with 400 mg/kg UP360 + D-gal had a significantly higher percentage of circulating T cells (CD3+CD45+; *P*=0.01; Figure 5(a)), Helper T cells (CD3+CD4+; *P*=0.009; Figure 5(b)), and natural killer cells (CD3−NKp46+; *P*=0.01; Figure 5(d)) than the D-gal + vehicle group, indicating that UP360 increased expansion or differentiation of the disclosed cell types in response to the influenza vaccination (Table 2). The changes observed for the normal control mice without the D-gal were not statistically significant.

### 3.4. Effect of UP360 on Circulating Immune Cell Populations in Nonimmunized Mice

**Figure 6:**
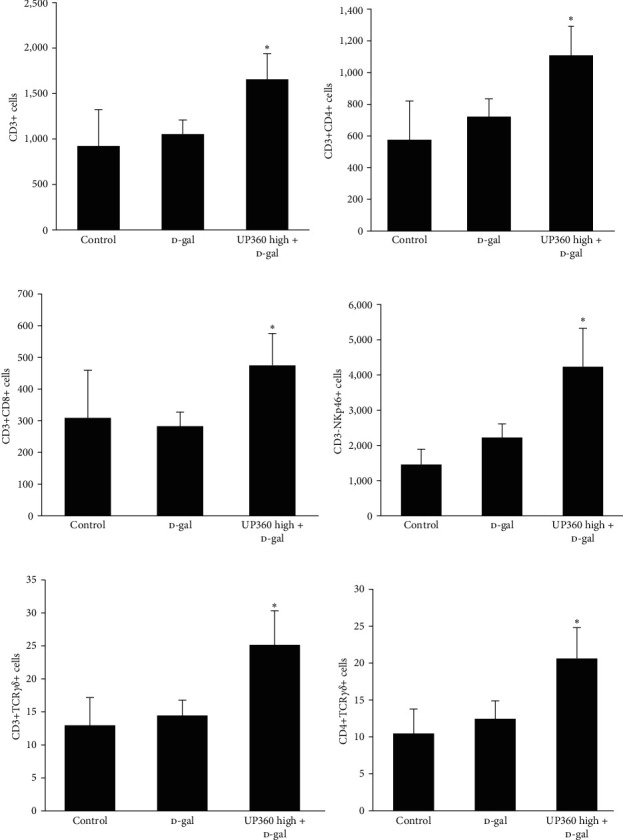
Effect of UP360 on immune cell expansion in nonimmunized mice. Mice (*n* = 8) were injected with D-galactose at 500 mg/kg subcutaneously daily for 9 weeks to induce accelerated aging. Mice were treated with UP360 at 400 mg/kg orally starting at week 5. Necropsy was carried out at the end of week 9. Flow cytometry data are reported as mean ± SD. (a) T cells; (b) helper T cells; (c) cytotoxic T cells; (d) natural killer cells; (e) Gamma–delta T cells; (f) Gamma–delta Helper T cells.  ^*∗*^*P* ≤ 0.05 vs D-gal control.

Compared to the D-gal nonimmunized group, the nonimmunized 400 mg/kg UP360 + D-gal group had significantly higher CD3+ cells per *μ*L of whole blood (*P*=0.02; Figure 6(a)). The same increases were seen for CD3+CD4+ Helper T cells (*P*=0.03; Figure 6(b)), and CD3+CD8+ Cytotoxic T cells (*P*=0.03; Figure 6(c)). These findings indicated higher levels of Helper T cells, Cytotoxic T cells, and T cells in general in the UP360 + D-gal nonimmunized group compared to the D-gal only group, which may have indicated better immune surveillance and “readiness” in the UP360-treated group. Similarly, a significant increase in the CD3−NKp46+ cells natural killer cells (*P*=0.04; Figure 6(d)), CD3+TCR*γδ*+ Gamma–delta T (*P*=0.02; Figure 6(e)), CD3+CD4+TCR*γδ*+ Gamma–delta Helper T (*P*=0.04; Figure 6(f)) were observed for the nonimmunized 400 mg/kg U360 + D-gal group compared to the vehicle-treated nonimmunized D-gal group. The significant increase in the NK and Gamma–delta T cells could indicate increased mucosal protection activity of the Aloe-based composition.

### 3.5. Effect of UP360 on Humoral Response

**Figure 7:**
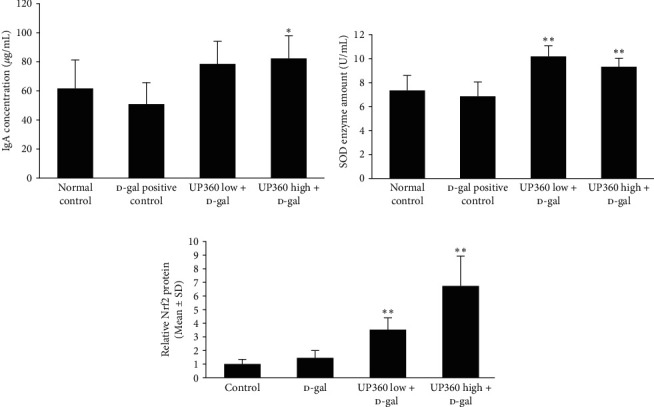
Effect of UP360 on humoral immunity and oxidative stress markers. Mice (*n* = 10) were injected with D-galactose at 500 mg/kg subcutaneously daily for 9 weeks to induce accelerated aging. Mice were treated with two doses of UP360 (200 or 400 mg/kg) orally starting at week 5 and immunized at the end of week 7 (start of week 8) with 3 *μ*g of Fluarix quadrivalent vaccine. Necropsy was carried out 14 days after vaccination at the end of week 9. Serum ELISA data for immunoglobulin A (Figure 6(a)) and SOD (Figure 6(b)) and spleen homogenate (Figure 6(c)) data for Nrf2 are reported as mean ± SD.  ^*∗*^*P* ≤ 0.05 vs D-gal control;  ^*∗∗*^*P* ≤ 0.001 vs D-gal control.

Serum was collected at the end of the study and assessed for markers of humoral immunity. Marked changes were observed only for the immunized mice. The D-gal + 200 mg/kg UP360-treated immunized mice had a trend toward higher levels than the vehicle treated D-gal group (*P*=0.06) in serum IgA levels, whereas the D-gal + 400 mg/kg UP360 immunized mice group had significantly higher serum IgA than the D-gal group (*P*=0.03; Figure 7(a)). The increase in the IgA level was very minimal for the immunized normal control mice without the D-gal when compared to the vehicle treated D-gal group. This increase in serum IgA indicates better adaptive immune function in the UP360-treated groups.

### 3.6. Effect of UP360 on Antioxidation Markers

Serum samples from the immunized mice were tested for levels of SOD to evaluate the general antioxidation capacity of the immunized mice. Statistically significant increases in the level of SOD were observed for both the 200 (*P*=0.003) and 400 mg/kg (*P*=0.01) UP360-treated immunized mice compared to the vehicle treated D-gal mice (Figure 7(b)).

A dose-correlated increase in Nrf2 protein level was observed for immunized mice treated with UP360. Spleen homogenates were run on SDS-PAGE and blotted for Nrf2. Nrf2 band intensity was measured by densitometry and normalized to the *β*-actin loading control. Semiquantitation of the protein was compared for both the groups and it was found that the immunized 200 mg/kg UP360 + D-gal (*P*=0.01) and 400 mg/kg UP360 + D-gal (*P*=0.004) groups had significantly higher Nrf2 than the D-gal alone, indicating an increase in antioxidation pathway activation in the UP360 groups (Figure 7(c)).

## 4. Discussion

Aging is a convoluted degenerative process wherein poor immune response isone of the most observed changes. With the notion that natural products containing diverse bioactives with multiple mechanisms of action could result in a balanced immune response, we tested an Aloe-based composition, UP360, in a chemically-induced accelerated aging mouse model.

Individual constituents of UP360 have showed immunomodulatory, antioxidant and anti-inflammatory activities *in vitro* and *in vivo*. Previous studies have shown the immune modulation activity of polysaccharides from Aloe and Poria and polyphenols from Rosemary. For example, *Poria cocos* polysaccharides (PCP) were found to increase the number of circulating cytotoxic T-lymphocyte [21], improve macrophage phagocytosis, thymus index and spleen index [12], increase serum levels of IgA, IgG, and IgM [22], decrease reactive oxygen species (ROS) and increase SOD [23] and have been used as an adjuvant in the rabies and hepatitis B vaccines [24, 25]. Similarly, immune modulation activity of Aloe polysaccharide has been reported, including inducing dendritic cell maturation [26], improving T cell proliferation, increasing antibody production and mitigating immunosuppression [27], stimulating Peyer's patch cells for cytokine production and restoring IgA production [28]. Likewise, anti-inflammatory and antioxidation properties of RA has been reported. RA was found to significantly reduce levels of the proinflammatory cytokines: IL-6, TNF-*α*, IFN-*γ*, MCP-1, and high mobility group box-1 protein (HMGB-1) [19, 29], reduce NF-*κ*B and MAPK activation [30], reduce mRNA levels of TNF-*α*, IL-6, IL-1*β*, increase mRNA level of IL-10, increase activity of the glutathione peroxidase (GPx) and SOD enzymes and inhibit the NF-*κ*B and JNK MAPK pathways [31]. As such, we hypothesized that the Aloe-based composition UP360, standardized for polysaccharides and polyphenols from medicinal plants, would produce a balanced immune response and tested it in a D-galactose-induced model known to mimic real time immunosenescence. This is the first time that these medicinal plants were standardized to yield a composition such as UP360.

The merit of combining three medicinal plant extracts (Aloe extract, Poria extract, and Rosemary extract) in immune regulation was demonstrated in a PBMC cytotoxicity assay. We utilized PBMCs isolated from whole blood to test UP360 and its constituent plant extracts to determine if they promoted the cytotoxic activity of PBMCs against tumor cells. Activated natural killer (NK) cells, which make up 10%–30% in the PBMC population primarily conduct cytotoxic activity [32]. UP360-treated PBMCs exhibited a statistically significant increase in cytotoxicity of K562 lymphoblasts beyond the cytotoxicity observed in the negative control. Its constituents also increased PBMC cytotoxicity, with the increased cytotoxicity effect of the three constituents equaling the total cytotoxicity observed in UP360-treated PBMCs for an additive effect.

Repetitive subcutaneous administration of D-galactose into mice produces a poor immune response, resembling changes that occur in the normal aging process. A higher thymus index corresponds to a stronger immune response [33]. The thymus indices for the normal control group and both UP360 + D-gal treatment groups were found to be significantly higher than the D-gal group, demonstrating the protection of this immune organ from senescence by the Aloe-based composition, which could lead to increased immune cell production and maturation. Substantiating our findings using this model, Wei et al. [8] have demonstrated enhanced proliferation of immune cells as a result of amelioration of thymus senescence and prevention of involution.

Significant changes in humoral immunity among the immunized groups were observed in this study. The UP360 + D-gal groups had increased serum IgA antibodies compared to the D-gal + vehicle group. This increased level of IgA in the serum indicated that the mucosa achieved a higher level of immune protection because of UP360 treatment, highlighting the significance of the composition in providing a mucosal shield through augmenting the humoral response. Supporting our finding, elevating the diminished levels of serum immunoglobulin levels was reported as among the factors found to mitigate D-gal-induced immune function impairment *in vivo* [34].

Administration of the Aloe-based composition, UP360, resulted in enhanced innate and adaptive immunity. In measuring the white blood cells in whole blood from the different groups and expressing changes as percentages of cell populations, significant differences among the immunized mouse groups were observed favoring the Aloe-based composition. CD3+ T cells, CD4+ Helper T cells, NKp46+ NK cells, and TCR*γδ*+ Gamma–delta T cells were all increased in the immunized UP360 + D-gal group compared to the immunized D-gal only group. These data indicated that the Aloe-based composition, UP360, aided in expansion of immune cell populations, resulting in higher percentages of innate and adaptive immune cells as critical mediators of immune homeostasis. These observations suggest the need for further exploration of the application of the Aloe-based composition as a possible adjuvant at the time of seasonal flu vaccination for the elderly. Expressed as total cells per *μ*L of whole blood, profound differences among the nonimmunized mouse groups were observed. The 400 mg/kg UP360 + D-gal group had increased CD3+ T cells, CD4+ Helper T cells, CD8+ Cytotoxic T cells, NKp46+ NK cells, TCR*γδ*+ Gamma–delta T cells, and CD4+TCR*γδ*+ Helper Gamma–delta T cells than the immune-compromised D-gal only group. These data implied that the Aloe-based composition, UP360, primed the inactivated immune system and caused expansion of immune cell populations, which could indicate increased immune “readiness” in the nonimmunized mice.

The activation and expansion of NK cells and TCR*γδ*+ Gamma–delta T cells is among the key modes of immunomodulation property of the Aloe-based composition, UP360. The marked increase in NK cells in the *in vivo* and *in vitro* studies coupled with the expansion of the Gamma–delta (*γδ*) T cells in the D-gal model were clear indications that the composition has a significant impact on innate immunity modulation, suggesting its potential usage for a rapid immune response and immune priming activity for overall enhanced immune surveillance and alertness [35–37].

Antioxidant enzymes and biomarkers were examined in order to surveil antioxidation pathways in the D-galactose-induced immune senescence model. We found an increase in SOD enzyme in mouse sera from immunized UP360 + D-gal groups compared to D-gal alone. Similarly, Nrf2, a transcription factor involved in activating antioxidation pathways in response to inflammation and prolonged oxidative stress [38], was significantly increased in spleen homogenates from the UP360 + D-gal groups compared to D-gal alone. These findings indicated that the Aloe-based composition enhanced antioxidation pathways that enabled the animals to neutralize free radicals better than the untreated aging animals.

The immune cell priming and expansion activities from the Aloe-based composition, UP360, are believed to be the result of the immune modulatory effects of the natural bioactives polysaccharide and polyphenols. UP360 constitutes intermediate-sized Aloe polysaccharides and mushroom *β*-glucan with a *β*-(1→3) backbone and *β*-(1→6)-linked side chains, which are known to be highly immunogenic [39–41]. Mammalian cells do not contain *β*-glucan or do not produce enzymes to process it, causing the indigestible *β*-glucan to be considered as a pathogen-associated molecular pattern by the body's defense mechanism [42]. As a result, it has been reported that in addition to being digested by the gut microbiome, *β*-glucan could also be sensed by the pattern recognizing receptors (e.g., dectin-1) on the surface of intestinal innate immune cells to activate innate and adaptive immune responses [43]. At times, it could be compartmentalized, digested, and released as small fragments by the intestinal macrophages to the systemic circulation to activate other immune cells and illicit a generalized immune response [44]. It has also been reported that polysaccharides could also be transported by the microfold cells of the gut intestinal epithelial cells in addition to being directly trapped and presented by the dendritic cells to the gut associated lymphoid tissue (Peyer's patches) immune cells for a subsequent immune cell priming and immune activation [45].

## 5. Conclusion

Altogether, significant changes in immune cell populations, antioxidative stress pathways, and protection of immune organs were observed in the animals treated with the Aloe-based composition, UP360. The increased immune system priming and activation favored the composition in a reversion inclined to the phenotype of the normal mice. These findings demonstrated the ability of the Aloe-based composition to aid in activating and maintaining homeostasis of the immune system, both during active infections and as a preventive measure to prime the immune system for better surveillance and alertness against a possible pathogen invasion.

## Figures and Tables

**Table 1:** Immune cell markers.

Cell type	CD45+	CD3+	CD4+	CD8+	CD49b+	NKp46+	Ly6C+	B220+	TCR*γδ*+
Lymphocytes	+	+	−	−	−	−	−	−	−
T cells	+	+	−	−	−	−	−	−	−
Helper T cells	+	+	+	−	−	−	−	−	−
Cytotoxic T cells	+	+	−	+	−	−	−	−	−
Natural killer cells	+	−	−	−	+	+	−	−	−
Granulocytes	+	−	−	−	−	−	+	−	−
B cells	+	−	−	−	−	−	−	+	−
Gamma–delta T cells	+	+	−	−	−	−	−	−	+
CD4+ gamma–delta T cells	+	+	+	−	−	−	−	−	+
CD8+ gamma–delta T cells	+	+	−	+	−	−	−	−	+

**Table 2:** Immune cell proportion (% of total live cells).

	Nonimmunized normal control	Immunized normal control	Nonimmunized D-gal group	Immunized D-gal group	Immunized 200 *µ*g/mL UP360	Nonimmunized 400 *µ*g/mL UP360	Immunized 400 *µ*g/mL UP360
Lymphocytes	86.6	67.8	89.0	90.7	73.9	89.1	81.0
T cells	26.0	11.4	16.4	12.8	15.1	18.9	15.4
Helper T cells	17.1	7.56	11.3	7.65	9.83	13.0	10.3
Cytotoxic T cells	7.90	2.98	4.39	4.32	4.53	5.03	4.25
Natural killer cells	32.8	33.3	32.7	25.7	29.5	49.0	35.0
Granulocytes	13.4	19.3	27.6	31.8	16.1	17.7	17.8
B cells	37.5	31.6	35.8	35.7	34.6	43.7	38.2
Gamma–delta T cells	0.39	0.23	0.21	0.24	0.32	0.30	0.31
CD4+ gamma–delta T cells	0.33	0.18	0.17	0.21	0.27	0.25	0.29
CD8+ gamma–delta T cells	0.04	0.02	0.03	0.04	0.05	0.03	0.05

## Data Availability

Conclusions were based on data depicted in the body of the manuscript. The data used to support the findings, interpretation, and conclusion of this study are included within the article. Additional data could be requested through the corresponding author.
